# The reliability of suicide statistics: a systematic review

**DOI:** 10.1186/1471-244X-12-9

**Published:** 2012-02-14

**Authors:** Ingvild Maria Tøllefsen, Erlend Hem, Øivind Ekeberg

**Affiliations:** 1 Department of Acute Medicine, Oslo University Hospital Ullevaal, PO Box 4956, Nydalen NO-0424 Oslo, Norway; 2 Department of Behavioural Sciences in Medicine, Institute of Basic Medical Sciences, Faculty of Medicine, University of Oslo, PO Box 1111, Blindern NO-0317 Oslo, Norway

## Abstract

**Background:**

Reliable suicide statistics are a prerequisite for suicide monitoring and prevention. The aim of this study was to assess the reliability of suicide statistics through a systematic review of the international literature.

**Methods:**

We searched for relevant publications in EMBASE, Ovid Medline, PubMed, PsycINFO and the Cochrane Library up to October 2010. In addition, we screened related studies and reference lists of identified studies. We included studies published in English, German, French, Spanish, Norwegian, Swedish and Danish that assessed the reliability of suicide statistics. We excluded case reports, editorials, letters, comments, abstracts and statistical analyses. All three authors independently screened the abstracts, and then the relevant full-text articles. Disagreements were resolved through consensus.

**Results:**

The primary search yielded 127 potential studies, of which 31 studies met the inclusion criteria and were included in the final review. The included studies were published between 1963 and 2009. Twenty were from Europe, seven from North America, two from Asia and two from Oceania. The manner of death had been re-evaluated in 23 studies (40-3,993 cases), and there were six registry studies (195-17,412 cases) and two combined registry and re-evaluation studies. The study conclusions varied, from findings of fairly reliable to poor suicide statistics. Thirteen studies reported fairly reliable suicide statistics or under-reporting of 0-10%. Of the 31 studies during the 46-year period, 52% found more than 10% under-reporting, and 39% found more than 30% under-reporting or poor suicide statistics. Eleven studies reassessed a nationwide representative sample, although these samples were limited to suicide within subgroups. Only two studies compared data from two countries.

**Conclusions:**

The main finding was that there is a lack of systematic assessment of the reliability of suicide statistics. Few studies have been done, and few countries have been covered. The findings support the general under-reporting of suicide. In particular, nationwide studies and comparisons between countries are lacking.

## Background

In recent decades, research on suicide and suicidal behaviour has expanded. Preventing suicide and reducing suicidal behaviour are important targets of the World Health Organization (WHO) [1]. The WHO has estimated that, worldwide, about one million people die by suicide every year, representing a global annual suicide rate of 16 per 100,000 people [2]. In addition, the suicide attempt rate is about 10-15 times more frequent than the suicide rate [3,4]. These suicide estimates are based on national mortality statistics, with suicide rates ranging from no suicides per 100,000 people per year in countries such as Egypt, Haiti and Honduras, to more than 30 suicides per 100,000 people per year in Belarus, the Russian Federation and Lithuania [5].

Most countries in the industrialized world started to register the cause and manner of deaths at the end of the 19th or the beginning of the 20th century. WHO member states use the International Classification of Diseases (ICD) to classify diseases and death certificates. The first edition, known as the International List of Causes of Death, was adopted in 1893. Even with this long tradition of classification, it is difficult to compare statistics between countries and periods because of differences between countries in methods of classification and registration, and because the manner of registration has changed over time.

National mortality registers have been used in the past few decades for surveillance and research on suicide, and can be used to examine the effects of preventive strategies and priorities in health policy. Epidemiological or socio-demographic theories about suicide and the effects of intervention depend on reliable suicide statistics. Many scientists have pointed out this challenge [6,7], but to our knowledge, no systematic research has been done in this field.

The aim of this study was to assess the reliability of suicide statistics through a systematic review of the international literature.

## Methods

### Search strategy

The first author (IMT) searched for relevant literature up to June 2009 in five databases: EMBASE (from 1980), Ovid Medline (from 1950), PsycINFO (from 1806), the Cochrane Library (from 1993) and PubMed (from 1950). The search strategy included subject headings/MeSH terms and free text. MeSH headings and free text included the terms "suicide" combined with "reliability", "test reliability", "validity", "test validity", "reproducibility", "reproducibility of results", "cause of death" and "death certificates". The search was restricted to humans. The search was not restricted by language, publication type or study design (Additional file 1). In addition, related studies and reference lists of identified studies were screened. Update searches were performed in October 2010, but no new studies were found.

### Study selection

All abstracts identified using the above search strategy were reviewed. The first author (IMT) excluded studies that were obviously irrelevant to this review. Then, the three authors screened the abstracts for relevancy, and independently reviewed the abstracts of all potentially relevant studies. Studies were included if they met the inclusion criteria of having the aim of studying the reliability of suicide statistics, and being published in English, German, French, Spanish, Norwegian, Swedish or Danish.

**Figure 1:**
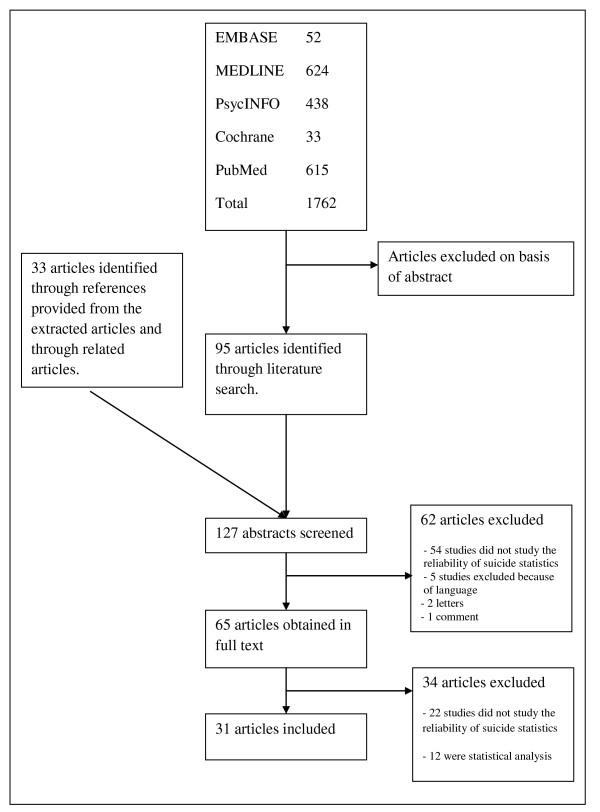
**Study selection flow chart**.

Studies were excluded if they were case reports, editorials, letters, comments, statistical analyses or studies only presented as abstracts. Any disagreements or differences in the extracted data between authors were resolved through consensus. If there were any doubts, we included the abstract and read the full text of the article. After excluding articles based on the abstracts, the authors performed a second, stricter screening by examining full-text reports of the remaining records. Disagreements regarding the eligibility were resolved through consensus. Reasons for exclusion were documented. We included all studies on the reliability of suicide statistics. The process of study inclusion is shown in Figure 1.

**Figure 2:**
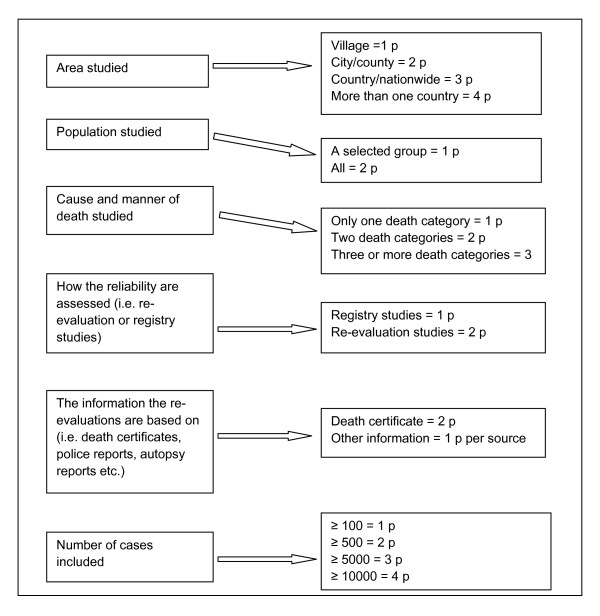
**Assessment of methodological quality**.

We did not attempt a meta-analysis because of methodological differences across studies. We assessed the methodological quality of the included studies using six criteria; area studied, population studied, cause and manner of death studied, how the reliability were assessed, the information the re-evaluations were based on, and number of cases included. The criteria of assessment of methodological quality are shown in Figure 2.

### Three ways of assessing the reliability of suicide statistics

There are three ways to assess the reliability of suicide statistics: re-evaluation studies, registry studies and statistical analyses. Re-evaluation studies are studies where the manner and cause of death were re-evaluated. Registry studies are studies where two cause-of-death registers are compared. Statistical analyses are studies where the suicide rate is calculated by adding other categories of manner and cause of death, usually undetermined deaths, open verdicts, and unintentional poisoning and drowning. We excluded statistical analyses from this review because the choice of which other categories of manner or cause of death to include often relies on registry or re-evaluation studies.

### Analyses

Some studies did not calculate the percentage of under-reporting, but calculated a study suicide rate and compared it with the official suicide rate. In those studies in which it was possible to estimate the percentage of under-reporting, we calculated the percentage by dividing the difference between the study and official suicide rates by the official suicide rate (under-reporting = (study suicide rate-official suicide rate)/official suicide rate).

## Results

### Study selection

The primary search yielded 127 potential studies. Of these, 31 studies [8-38] met the inclusion criteria, with a population of 46,401 cases in the final review. Three of the studies did not describe the exact number of cases, and were excluded from the total number of cases. Of the 96 excluded articles, 76 were excluded because they did not study the reliability of suicide statistics, 12 were statistical analyses, two were letters, one was a comment and five were excluded because of language (Romanian, Portuguese, Czech, Serbian, Dutch).

### Study characteristics

#### Methodologies and sample size

Of the 31 included studies, 23 were re-evaluation studies, with a total population of 11,795 cases (range: 40-3,993). Six studies were registry studies, with a total population of 31,436 cases (range: 195-17,412). The remaining two studies were combined registry and re-evaluation studies, with a total population of 3,170 cases (442 and 2,728 cases). Eight re-evaluation studies evaluated a nationwide representative sample, although these samples were limited to suicide within subgroups. Three registry studies evaluated a nationwide sample. Only two studies compared data from two or more countries. Characteristics of the included studies are presented in Table 1.

**Table 1:** Characteristics of the included studies by type of study and publication year

Study	Publication-year	Study period	Area studied	Population	Cause and manner of death studied	Method of sampling	Number of cases(n)	Results
**Re-evaluation studies**								

Tsung-Hsueh, Lu et al. [37]	2006	1.1-31.12.2002	Taiwan (nationwide)	All	S, U	Re-evaluated DC	3993	Fair quality of mode of death certification (MOD).

Carr J.R. et al. [36]	2004	1998-1999	USA (nationwide)	US Military	A*, U, H and deaths occurred within 30 days of retirement	Re-evaluated DC, AR, TR, CR, MJ and investigative agency reports	1844	Concluded 21% underestimation.
								Found 17% underreporting, and additional 4% of deaths that were suspicious for suicide.

Ahlm, K. et al. [33]	2001	1999	Sweden (nationwide)	All	Traffic deaths	Re-evaluated AR, DC, MJ and PR	580	¶ 1.5% under-reporting.
								Found 3% (18 of 580) suicides among the officially registered accidental traffic deaths.

Sampson, H.M. and Rutty, G.N [31]	1999	1992-1997	South Yorkshire (West), England	All	S, OV	Re-evaluated MJ, AR, and SN	295	Official national data may under-report the annual suicide rate by over 20%.

Ohberg, A. et al. [30]	1998	1.4.1987 - 31.3.1988	Finland (nationwide)	All	U	Re-evaluated MJ and psychological autopsies.	139	Undetermined deaths resembled suicides and appeared to reduce the suicide rate by 10%

Connolly, J.F. and Cullen, A [28]	1995	1978-1992	County Mayo, Ireland	All	S, A**, U	Re-evaluated DC, MJ	220	35% deaths were miscoded (n = 56) or unregistered (n = 16).

O'Donnell, I. and Farmer, R [29]	1995	A 5-year study period	London (England)	All	S (deaths on the London Underground railway system)	Re-evaluated. Records from LUL and the British Transport Police. Prospective design	242	Substantial underestimation of the true number of suicides.
								Verdicts other than suicide were returned on 25% and 50% of the women and men, respectively.

Scott, K.W.M [26]	1994	1976-1990	The borough of Wolverhampton (250.566) (England)	All	S	Re-evaluated AR	394	¶¶ 69% under-reporting.
								Estimated suicide rate of 10.5/100,000 per year. The official suicide rate were 6.2/100 000 per year. The coroners only reflected 59% of the probable true suicide rate.

Allebeck, P. et al. [24]	1991	1969-1983	Sweden (nationwide)	Males conscripted for military service in 1969-70	CO, S, U, TD	Re-evaluated DC, PR, MJ, TR and AR in a cohort of 50.465	322	High accuracy. Findings indicate an underreporting of suicide in cases of poisoning and other undetermined cases.

Rodriguez-Pulido, F. et al. [23]	1991	1977-1983	Canary Islands (Spain)	All	VD, ND	Re-evaluated AR, SN, TE and JP	775	¶¶ 104% under-reporting.
								Lack of validity and reliability of official figures of suicide. Recorded 8.1/100,000 suicides per year. The official published statistics recorded 3.98/100,000 per year for the same period. This represents 49% of which was found in this study.

Walsh, D.et al. [22]	1990	1978-1987	County Kildare, County Dublin and Dublin city (Ireland)	All	S, A, U	Re-evaluated CR	-	¶¶ 5% under-reporting.
								Reflecting the suicide rate accurately.
								Estimated 5.9/100,000 suicides per year. The official suicide rate were 5.6/100,000.

Huusko, R. and Hirvonen, J [20]	1988	1981	Oulu, Finland	All	S, A, U	Re-evaluated PR, AR and MJ	283	The official figure for suicides could be as much as 18.9% too low.

Ekeberg, Ø. et al. [17]	1985	24.5.1978 - 26.4.1981	Norway (except Hordaland and Sogn og fjordane)	All	DI, D	Re-evaluated AR	210	Underregistration of 10%

Malla, A. and Hoenig, J [16]	1983	1974-1978	Newfoundland (Canada)	All	S, CO	Re-evaluated DC and AR	104	¶¶ 12% under-reporting.
								Study suicide rate of 4.25/100,000 suicides per year, compared to the official rate of 3.8/100,000 per year.

Clarke-Finnegan, M. and Fahy, T. J [15]	1983	1978	Galway, Ireland	All	All deaths except natural deaths and TD	Re-evaluated AR	410	¶¶ 126% under-reporting.
								A minimum true rate of suicide was 13.1/100,000. The officially reported suicide rate was 5.8/100,000.

De Faire, U. et al. [13]	1976	1961-1973	Sweden (nationwide)	Twins born in 1901-1925	All deaths	Cohort of twins. Re-evaluated MJ, AR and PR	1156	Mortality data on suicide are fairly valid for use in epidemiological studies and mortality statistics with regard to suicides.

McCarthy, P. D. and Walsh, D [11]	1975	1964-1968	Dublin (Ireland)	All	All deaths investigated by Dublin city and county coroners	Re-evaluated CR, MJ	210	¶¶ 279% under-reporting.
								Official suicide rate of 1.4 per 100,000 and study-rate of 5.3 per 100,000.

Ovenstone, I.M.K [9]	1973	Oct.1969 - March 1971	Edinburgh (Scotland)	All	S, A, U and gas poisonings	Re-evaluated MJ	214	Suicide rate was underestimated by approximately 50% by the Crown Counsel.
								The Crown counsel under-reporting suicide by 40.6% and the Scottish Registrar General 32%.

Litman, R.E. et al. [8]	1963	1959-1960	Los Angeles County	All	Equivocal suicides	Re-evaluated PR, AR and interviewed survivors of the deceased.	100	Significant underreporting.

**Combined re-evaluation and registry studies**								

Cantor, C. et al. [32]	2001	1990-1995	Queensland, Australia	All	Suicide beyond no reasonable doubt, probable and possible suicide	Registry study and re-evaluated PR and AR	2728	5.5% more suicides registered in QSR than the official ABS count for the period.
								141 of the deaths coded as probable/possible suicides (675) by the QSR were rejected as being deaths other than suicide - usually accidental overdoses.

Thorslund, J et al. [21]	1989	1977-1986	Greenland	All	S, CO	Registry study and Re-evaluated DC and PR	442	The official statistics are generally reliable.

**Registry studies**								

Elnour, A.A. and Harrison, J [38]	2009	July 2000 - the end of 2005	Australia (nationwide)	All	S	Registry study	12786	About 8% underestimation

Lahti, R.A. and Vuori, E [35]	2003	1997	Finland (nationwide)	All	DPD; A, S, U	Registry study	500	Fairly good agreement. 98.5% agreement

Lindeman S.M. et al. [27]	1995	1986-1991	Finland (nationwide)	All	S, U, A***	Registry study	17 412	Reliable enough. 97% coverage.

Van de Voorde, H. et al. [25]	1993	1981-1984	Leuven (Belgium)	All	S	Registry study	323	Incidence reporting bias of 4-8%.
								311 suicides found in one registry (PPO) and 323 suicides in the other registry (NIS).

Gary Hlady, W. and Middaugh, J.P [19]	1988	1983-1984	Alaska	All	S	Registry study	195	Severely under-recorded suicides.

Marshall, D.L. and Soule, S [18]	1988	1979-1984	49 predominantly Native villages in southwest Alaska	All	VD; S, A, U, H	Registry study	220	¶¶405% under-reporting.
								Native suicide rate 36.9/100,000 (n = 38), compared to official suicide rate 7.3/100,000 (n = 5).

**"Other re-evaluation studies"**								

Joseph, A. et al. [34]	2003	1994-1999	Kaniyambadi region, southern India	All	-	Verbal autopsies	-	Mean suicide rate was 95.2/100,000 (range 83.7-106.3/100,000) - nine times the national average

Warshauer, M.E. and Monk, M [14]	1978	1968-1970	An area of New York City; East Harlem in Manhattan and three districts in the South Bronx. (USA)	All	S, "assigned" suicide	Comparing published Health Department suicide rates with medical examiner records	-	Suicides among the black population were underestimated by 80% and those among whites by 42%. Suicide rates were greatly affected by the change in revision of the ICD.

Atkinson, M.W. et al. [10]	1975	-	England and Denmark (nationwide)	All	Probable suicide	Compared suicide ascertainment procedure between Denmark and England.	40	Danes consistently report more suicides than do the English coroners

Ross, O. and Kreitman, N [12]	1975	-	England, Wales, Scotland (nationwide)	All	S, U, OV	A two-way exchange of case records	264	The results strongly suggest that the two sets of officials share roughly the same criteria.

*A *accident, *AD *all deaths, *AR *autopsy-/forensic-/post-mortem reports, *CO *controversial/unclear/no cause of death reported, *CR *coroner/medical examination records, *D *drowning, *DC *death certificates/report, *DI *deaths from intoxication, *DPD *drug poisoning deaths, *H*: homicide, *JP *judicial proceedings, *MJ *medical-/hospital journal/-report, *ND *natural death where insufficient medical information exists to permit the issuing of a death certificate stating that the cause of death was a natural one. *OV *open verdict, *PR *police reports, *S *suicide, *SN *suicide note, *TE *testimonies of relatives and witnesses, *TD *traffic deaths, *TR *toxicology reports, *U *undetermined, *VD *violent deaths, A* accidents by handgun, overdose, asphyxia, drowning and falling. A** road traffic accidents, fires in domestic dwellings and drownings were excluded. A*** motor vehicle accident, drowning, poisoning

¶ The authors have calculated the under-reporting: Found 18 "new suicides", total 1219 suicide and suicide rate 13.76/100,000 in Sweden in 1999 (data from Socialstyrelsen)

¶¶ ("study suicide rate" - official suicide rate)/official suicide rate

#### Year and location of studies

The included studies were published between 1963 and 2009. Fourteen studies were published between 1963 and 1989, ten between 1990 and 1999 and seven after 2000. Twenty were from Europe, seven from North America, two from Asia, and two from Oceania.

#### Characteristics of the study population

Of the included studies, one re-evaluated the reliability of suicide statistics within the military system. Two studies examined the causes of death in cohorts: one of young males conscripted for military service and one of twins. The other studies included all deaths within defined time periods, locations or subgroups according to the manner of death. Some studies evaluated only suicides, whereas others included homicides, accidents and undetermined deaths.

#### Analysis of the included studies

The main conclusions of the studies varied, with findings ranging from fairly reliable suicide statistics to considerable under-reporting. Thirteen studies (42%) reported fairly reliable suicide statistics or under-reporting of 0-10%. Of the 31 studies from the 46-year period, 52% (16 of 31 studies) found more than 10% under-reporting, and 39% (12 of 31 studies) found more than 30% under-reporting or poor suicide statistics. A summary of the conclusins of the included studies are presented in Table 2.

**Table 2:** Brief summary table

Conclusions	Total number of studies (n)	Re-evaluation studies	Registry studies
0-10% under-reporting	7	4	3

Fairly reliable suicide statistics	6	3	3

11-30% under-reporting	4	4	0

> 30% under-reporting	10	9	1

Poor suicide statistics	2	1	1

The two combined re-evaluation and registry studies are in this table registered as registry studies.

The two studies were they exchanged data between countries (Ross O. et al. [12] and Atkinson M.W. et al. [10]) are not included in this table.

#### Analysis of methodological quality

When summarizing the quality scores three studies [24,33,36] got a sum score ≥15, representing good quality. One of these studies concluded fairly reliable suicide statistics, one 0-10% under-reporting and one concluded 11-30% under-reporting. Twenty-one studies got a sum score 10-14 [8,9,11-13,15-17,20-23,27-32,35,37,38] and seven studies sum score ≤9 [10,14,18,19,25,26,34]. Five of the studies with quality sum score ≤9 concluded > 30% under-reporting or poor suicide statistics, one concluded 0-10% under-reporting. The quality sum score and the conclusions of the studies are presented in Table 3

**Table 3:** Summary quality sum score

Conclusions	Quality sum score ≤ 9	**Quality sum score 10**-**14**	Quality sum score ≥ 15
0-10% under-reporting	1	5	1

Fairly reliable suicide statistics	0	5	1

11-30% under-reporting	0	3	1

> 30% under-reporting	4	6	0

Poor suicide statistics	1	1	0

The two studies were they exchanged data between countries (Ross O. et al. [12] and Atkinson M.W. et al. [10]) are not included in this table.

## Discussion

### Summary of main results

The main finding was that few studies on the reliability of suicide statistics have been done in recent years, and few countries have been covered. There were only two studies from Asia and none from Africa, where a large proportion of the global population resides. Thirteen of the 31 studies included in this review concluded with fairly reliable suicide statistics or under-reporting of 0-10%. Of the 31 studies from the 46-year period, 52% found more than 10% under-reporting, and 39% found more than 30% under-reporting or poor suicide statistics. Eleven studies evaluated a nationwide sample, and only two studies compared data from two or more countries. Only three studies got a good quality sum score. It is a trend that studies with high quality sum score concluded with fairly reliable suicide statistics or under-reporting of 0-10%, while studies with poorer quality sum score tends to conclude with more than 30% under-reporting or poor suicide statistics, but too few studies are done to make an absolute conclusion. We have put most emphasis on the studies with the best methodological quality. These studies support our main findings. We cannot make any conclusions about the reliability of suicide statistics based only on the lack of research. Theoretically, the reliability might be good in spite of the lack of studies. In countries with official suicide rates close to zero, one might argue that the reliability was good. It is important to study the reliability of suicide statistics, and since the data are very different in the various countries, we find it of importance to study both the validity and reliability. As there are few studies, and about half of them concluded with underreporting of suicide, we think that our main finding, that the reliability of suicide statistics is questionable and calls for more studies, is fear.

Reliability does not necessarily imply validity. A reliable measure is measuring something consistently, but it may not measure what it claims to do. A thorough forensic and psychological autopsy may be the most valid method to determine the cause of death, e.g. suicide. If the statistics consistently underreported suicide, the methods could be reliable enough to justify multivariate analyses of determinants and multivariate evaluations of interventions. In the present study, the causes of death were assessed by official statistics and the researchers had the intention of measuring the same phenomenon. Accordingly, we consider that comparing official suicide statistics and external assessments reflects both reliability and validity.

Studying the reliability of suicide statistics is a complex task. First, some suicides might have been missed in the administrative processes of national mortality statistics. Second, in some cases, determining the manner of death (i.e., suicide, accident, undetermined/open verdict, or natural death) requires subjective interpretation of the intention of the deceased. Different methodologies used in the included studies need to be considered, including the main difference between re-evaluation and registry studies, the variations in the cause and manner of deaths studied, the quality of the compared registers, the competence of the re-evaluators, the number of re-evaluations of each case and the information the re-evaluations are based on (i.e., death certificates, police reports, autopsy reports, etc.). One can imagine that a greater number of suicides could be found by also examining undetermined deaths/open verdicts and accidents. Some studies (statistical studies) have studied the under-reporting of suicide by comparing the suicide rate with the rate of deaths of undetermined intent [39,40], and in recent years, the UK has added injury/poisoning of undetermined intent and sequelae of intentional self-harm/event of undetermined intent to the official suicide rate, in the belief it will provide a more reliable suicide rate [41]. We excluded statistical studies in this review article, but in a national and longitudinal perspective these studies are important for indicating reliability of suicide statistics, and further, effects of suicide prevention. Studies included in this review were published in many different countries between 1963 and 2009. Hence, different editions of the ICD are used in these studies, which may also affect the results to a certain extent [42].

### Strengths and limitations

Some limitations of the present study should be considered. The search strategy, including literature search and reference list screening, was developed by one of the authors, and this search strategy may not have captured all relevant studies. Manually searching reference lists located 33 further studies not captured in the database searches. The selection of keywords and MeSH terms that were used may not have covered all published articles on the reliability of suicide statistics. The choice of databases also needs to be considered. The five selected databases may not have indexed all potential studies, and some relevant studies may not have been included. Medline is the largest component of PubMed, and both databases were selected in the present study because they do not have the same MeSH terms, and therefore more studies were found searching both http://www.nlm.nih.gov/pubs/factsheets/dif_med_pub.html. In retrospect, it is conceivable that using only one of these databases might have saved time, and we might have found more studies by selecting a different database or manually searched relevant journals. It is possible that our search did not identify all of the relevant original studies [43]; for example, the publications of national statistics bureaus are not indexed in the databases, but we are confident that our research strategy has been good enough to identify the majority of the relevant original studies. Even though we may have missed some studies, we find it unlikely that this would have changed our main conclusions. For practical reasons, only published studies were sourced, but it seems unlikely that publication status would be a source of bias in the present study.

One strength of this review is that related studies and all reference lists of the included studies were screened, minimizing the number of potentially missed studies. Another strength is that the three authors independently screened all abstracts and full-text articles, minimizing the chance of a relevant study being excluded.

### Future studies

The fact that few studies have been published in recent years, makes further studies clearly needed, particulary nationwide studies, studies in countries with low suicide rates and other under-investigated countries, and studies including comparisons between countries.

## Conclusion

There are only few studies on the reliability of suicide statistics, and based on those studies, we cannot draw firm conclusions about the reliability of existing suicide statistics. Few studies have been published in recent years. Nationwide studies in particular are lacking, and only two studies compared data between countries.

This systematic review conforms to the PRISMA statement [44].

## Competing interests

The authors declare that they have no competing interests.

## Authors' contributions

All authors contributed equally to the conception and design of the study, the selection of studies, and to the final version of the manuscript. IMT developed the search strategy, interpreted the data and drafted the manuscript. All authors read and approved the final manuscript.

## Pre-publication history

The pre-publication history for this paper can be accessed here:

http://www.biomedcentral.com/1471-244X/12/9/prepub

## Supplementary Material

Additional file 1 **Search terms**. Click here for file
