# Supplementary material for: The reliability of suicide statistics: a systematic review
Source: BMC Psychiatry. 2012 Feb 14;12:9. doi: 10.1186/1471-244X-12-9 (PMC3350416; doi:10.1186/1471-244X-12-9)
Supplement: Additional file 1 — Search terms. [file 1471-244X-12-9-S1.PDF]

Additional file: serach terms

<u>Medline (1950-2010)</u>

(Explode, Focus and include all subheadings in all MeSH-terms)

“Suicide” (MeSH) Limits to Humans

AND

“Reproducibility of results” (MeSH) OR “ Cause of death” (MeSH) OR “ Death certificates” (MeSH) OR “reliability” (keyword) OR “Validity” (keyword)

18.6.2009: 624 abstracts

8.10.2010: 780 abstracts
